# Major Pelvic Ring Injuries: Fewer Transfusions Without Deaths from Bleeding During the Last Decade

**DOI:** 10.1007/s00268-023-06897-7

**Published:** 2023-01-17

**Authors:** Giles L. Devaney, Seth M. Tarrant, Natasha Weaver, Kate L. King, Zsolt J. Balogh

**Affiliations:** 1 Department of Traumatology, John Hunter Hospital, Lookout Rd, Newcastle, NSW 2305 Australia; 2 School of Medicine and Public Health, University of Newcastle, Callaghan, NSW 2308 Australia

## Abstract

**Background:**

Pelvic fracture-associated bleeding can be difficult to control with historically high mortality rates. The impact of resuscitation advancements for trauma patients with unstable pelvic ring injuries is unknown. We hypothesized that the time elapsed since introduction of our protocol would be associated with decreased blood transfusion requirements.

**Methods:**

A level 1 trauma center’s prospective pelvic fracture database was reviewed from 01/01/2009–31/12/2018. All patients with unstable pelvic ring injuries initially presenting to our institution were included. Adjusted regression analysis was performed on the overall cohort and separately for patients in traumatic shock (TS). The primary outcome was 24 h packed red blood cell (PRBC) requirements. Secondary outcomes were 24 h plasma, cryoprecipitate, platelet and intravenous fluid (IVF) requirements, length of stay and mortality.

**Results:**

Patients with mechanically unstable pelvic ring injuries (*n* = 144, median [Q_1_–Q_3_] age 44 [28–55] years, 74% male) received a median (Q_1_–Q_3_) of 0 (0–4) units PRBC within 24 h, with TS patients (*n* = 47, 42 [28–60] years, 74% male) receiving 6 (4–9) units PRBC. There was no decrease in 24 h PRBC requirements for the overall cohort (years; IRR = 0.91, 95% CI 0.83–1.01; *p* = 0.07). TS patients had decreases in 24 h PRBC (years; IRR = 0.90, 95%CI 0.84–0.96; *p* = 0.002), plasma (IRR = 0.92, 95%CI 0.85–0.99; *p* = 0.019), cryoprecipitate (IRR = 0.88, 95%CI 0.81–0.95; *p* = 0.001) and IVF (IRR = 0.94, 95%CI 0.90–0.98; *p* = 0.004). There were 5 deaths (5/144, 3.5%) with no deaths due to acute hemorrhage.

**Conclusions:**

Over this 10-year period, there was no hemorrhage-related mortality among patients presenting with pelvic fractures. Crystalloid and transfusion requirements decreased for patients presenting with traumatic shock.

**Supplementary Information:**

The online version contains supplementary material available at 10.1007/s00268-023-06897-7.

## Introduction

Major pelvic ring injuries are frequently associated with hemodynamic instability and hemorrhage control can be challenging to achieve [1]. Management of mechanically and/or hemodynamically unstable pelvic trauma patients requires a multidisciplinary approach including rapid pre-hospital transport with non-invasive stabilization and initiation of hemostatic resuscitation. In hospital, focused imaging and early hemorrhage control with simultaneous resuscitation, and preferably definitive skeletal stabilization are associated with better outcomes [2]. Early hemostatic resuscitation is essential to minimize further blood loss and coagulopathy-associated mortality [3]. Currently, in addition to expeditious hemorrhage control, simultaneous resuscitation with balanced combination of packed red blood cells (PRBC), fresh frozen plasma (FFP), concentrated clotting factors (cryoprecipitate and/or prothrombin concentrate) and platelets is practiced [4]. While the benefits of early hemostatic resuscitation are well-documented, the trends in blood products used for the resuscitation of pelvic trauma patients and the impact on outcomes are largely unknown [5].

Our institution is a leader in early definitive pelvic stabilization for pelvic ring injuries in multi-trauma patients, a practice that has been in place since 2005 [6], with recent studies identifying time to definitive fixation for unstable and polytrauma patients has decreased [7]. Recent studies have also identified that the requirement for urgent hemorrhage control procedures such as pelvic angioembolization decreased after institutional policies of hemostatic resuscitation were implemented, without decreases in transfusion requirements [8]. Based on these findings, this study aims to describe the patterns of crystalloid and blood product administration over the last 10 years among major-trauma patients with unstable pelvic ring injuries. We hypothesized that PRBC and crystalloid requirements have decreased since the introduction of our modern hemostatic resuscitation protocols.

## Patients and methods

### Settings and inclusion criteria

This study was conducted at a University-affiliated Level 1 trauma center which receives over 4500 trauma admissions per year including approximately 500 patients with an injury severity score (ISS) greater than 15 [9]. Acute pelvic ring injuries are managed in accordance with ATLS principles and local pelvic fracture management guidelines [10].

All patients were identified through the hospital’s prospectively collected pelvic injury database. Consecutive patients presenting between 01/01/2009 and 31/12/2018 who required surgical stabilization of their pelvic ring injury were reviewed. All patients initially presenting to our institution were included. Patients who were transferred after being initially resuscitated at other centers were excluded due to the inability to accurately record blood and fluid transfusion volumes and adherence to our guidelines.

Patients were analyzed as an entire cohort with subgroup analysis for patients who were classified as being in traumatic shock (TS) on presentation. We pragmatically defined traumatic shock as physiological parameters prompting administration of ≥ 2 units of blood products in the pre-hospital or emergency setting, to isolate this cohort as patients presenting with hemodynamic instability due to tissue injury and hemorrhage. This approach reflects the acute clinical concern about bleeding and relevant clinician response to it.

This 10-year time period was chosen as a period where our institution’s protocol on the management of hemorrhage control for patients with hemodynamically unstable pelvic ring injuries and our massive transfusion protocol remained unchanged.

The STROBE statement guidelines were following to ensure proper reporting (Supplementary document 1).

Ethical approval was obtained from local clinical governance—Approval number AU2021209-25.

### Massive transfusion and hemorrhage control protocols

In the pre-hospital setting, patients are routinely stabilized with pelvic binders and receive hemostatic resuscitation (minimal crystalloid infusions and initiation of blood products such as PRBCs and FFP) en route by pre-hospital care providers. In the emergency department, if clinically indicated, an inpatient massive transfusion protocol (MTP) can be commenced. Our institution’s MTP was implemented in 2005 and is based on a 1: 1: 1 ratio of PRBC, FFP and cryoprecipitate with pooled platelets replacing cryoprecipitate in even-numbered packs and individual adjustments made based on blood gas results, point of care thromboelastography and physiological response [11]. Patients presenting with hemodynamic instability and pelvic ring injuries are resuscitated in the emergency department and reassessed during their first MTP. If their hemodynamics and blood gases are critical or not responding to hemostatic resuscitation, they are either transferred directly to angioembolization, or, if their physiological status makes it safe to do so, they are taken for trauma computed tomography (CT). If the fracture pattern is amenable for acute minimally invasive internal fixation, definitive fixation is performed immediately after imaging during ongoing resuscitation and other operative procedures. If major open surgery is required for definitive fixation of the pelvic ring, internal fixation is performed at the earliest safe timeslot preferably within 48 h from injury.

### Collected variables

Demographic data were collected prospectively for all patients on the trauma database (Age, Sex, Body region Abbreviated Injury Score [AIS], Injury Severity Score [ISS]).

Shock parameters (Systolic Blood Pressure, pH, Lactate and Base excess) and Glasgow Coma Scale (GCS) score were also prospectively collected. A retrospective review of patient files was conducted to determine intravenous crystalloid and blood transfusion requirements, both in the pre-hospital and emergency department/inpatient setting. Requirement for angiography and successful embolization was recorded. Time to transfusion was recorded for the TS subgroup.

Time to transfusion was defined as time (hours) from on-scene arrival of pre-hospital care providers until commencement of the first unit of blood products.

Head AIS was categorized as < 3 or ≥ 3 due to the difference in clinical management, consistent with previous definitions [12, 13]. GCS was categorized as < 9 or ≥ 9 due to the differences in clinical management [14, 15].

### Outcomes

The primary outcome was PRBC requirements within the first 24 h. Secondary outcomes include FFP, cryoprecipitate and platelet transfusion within the first 24 h, and intravenous crystalloid (IVF) administration within the first 24 h. Hospital length of stay (LOS), intensive care unit (ICU) LOS and mortality were recorded.

### Statistical analysis

Continuous data were assessed for distribution and presented as mean and standard deviation for normally distributed data or median with first and third quartiles for skewed data. Categorical data are presented as frequency count and percentage.

Analysis of primary and secondary outcomes was conducted for the entire cohort and repeated for the TS subgroup, a decision made a priori due to the dramatically differing physiology and treatment requirements for patients presenting in Traumatic shock.

The primary outcome, count of PRBC units within 24 h, was positively skewed with evidence of overdispersion. The relationship between year and outcome was analyzed via negative binomial regression, adjusted for potential confounding variables (Age, ISS category, Head injury severity category, GCS category, systolic blood pressure and base excess on arrival). Possible changes in demographic parameters and injury severity over the study period were accounted for in the multivariate model.

Time to blood transfusion (hours) was also identified a priori as a potential confounder and included in the multivariate analysis of the TS cohort. Secondary outcomes were analyzed similarly with either Poisson or negative binomial models after assessment of dispersion.

Results were presented as incidence rate ratio (IRR) of the outcome per year with 95% confidence interval. Patients with missing outcome data were not included in the analysis. A summary of missing outcome data is available in supplementary document 2, Table 1.

**Table 1:** Demographics and Injury descriptors of patients presenting with pelvic fractures requiring fixation

Variable	Level or statistic	TS (*n* = 47)	Overall (*n* = 144)
Age	Median (Q1, Q3)	42 (28, 60)	43.5 (28, 54.5)
Sex	Female	12 (26%)	37 (26%)
	Male	35 (74%)	107 (74%)
*Injury characteristics*			
ISS	Median (Q1, Q3)	38 (25, 45)	24 (17, 34)
Injury severity	ISS > = 16	45 (96%)	131 (91%)
	ISS < 15	2 (4.3%)	13 (9.0%)
Head injury	Yes	28 (60%)	58 (40%)
	No	19 (40%)	86 (60%)
Arrival GCS	Median (Q1, Q3)	14 (6, 15)	15 (14, 15)
GCS category	9–15	33 (70%)	127 (88%)
	3–8	14 (30%)	17 (12%)
*Mechanism of injury*			
MBC	*n* (%)	11 (24%)	38 (28%)
MVC	*n* (%)	23 (51%)	50 (37%)
Fall	*n* (%)	4 (8.9%)	21 (15%)
Other	*n* (%)	7 (16%)	27 (20%)
Fracture type			
Isolated Pelvic ring	*n* (%)	40 (85%)	122 (95%)
Pelvic and Acetabular	*n* (%)	7 (15%)	22 (15%)

*TS* – Traumatic Shock / *ISS*—Injury severity score / *GCS*—Glasgow Coma Scale / *MBC*—Motor Bike Crash / *MVC*—Motor vehicle crash

Statistical significance was set at 0.05. Statistical analysis was conducted with Stata v13.0 (StataCorp, College Station, TX).

## Results

### Patient selection and demographics

**Fig. 1:**
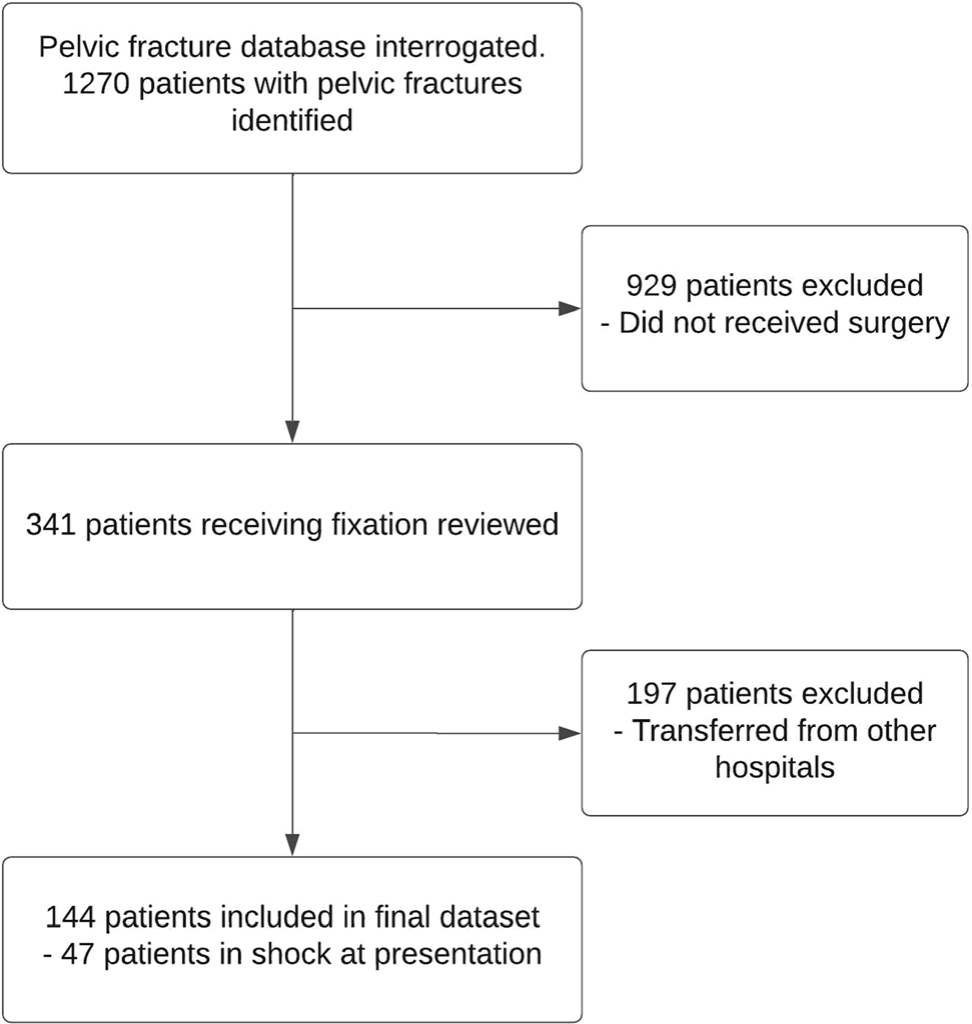
Inclusion flowchart of all pelvic fracture presentations over the study period

A total of 1270 patients presented with pelvic ring injuries were included into our institutional prospective pelvic fracture database throughout the 10-year period between January 1, 2009, and December 31, 2018, of these 144 patients met inclusion criteria and were included in this study (Fig. 1), with 47 patients meeting the criteria for the TS subgroup. A full list of patient demographics and injury parameters is available in Table 1. Physiological parameters at presentation, length of stay and mortality outcomes are illustrated in Table 2.

**Table 2:** Shock parameters, length of stay, angioembolization and mortality outcomes of patients presenting with pelvic fractures requiring fixation

Variable	Level of Statistic	TS (*n* = 47)	Overall (*n* = 144)
SBP	Mean (SD)	98 (84, 121)	112.5 (98, 128)
Base excess	Mean (SD)	− 5.2 (− 8.5, − 2.6)	− 2.3 (− 4.3, 0.1)
pH	Mean (SD)	7.3 (7.2, 7.3)	7.3 (7.3, 7.4)
Lactate	Mean (SD)	3.9 (2.7, 5.2)	2.4 (1.8, 3.8)
Required Angiography	*n* (%)	14 (30%)	14 (10%)
Embolized	*n* (%)	7 (15%)	7 (5%)
Hospital LOS	Median (Q1,Q3)	33 (20, 51)	22 (13.5, 42)
Required ICU	*n* (%)	43 (91%)	72 (50%)
ICU LOS	Median (Q1,Q3)	5 (2, 11)	0.5 (0, 4.5)
Mortality	*n* (%)	3 (6.4%)	5 (3.5%)

*TS* – Traumatic Shock / *SBP*—Systolic Blood Pressure / *LOS*—Length of Stay (days) / *ICU*—Intensive Care Unit

### Transfusion outcomes

#### 24 h PRBC requirements

**Fig. 2:**
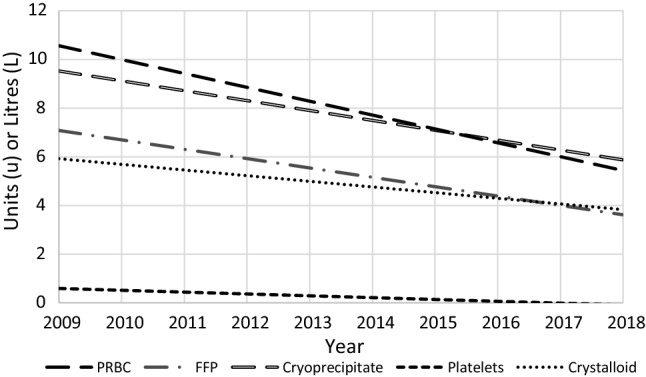
Blood and crystalloid administration per year for patients presenting in Traumatic Shock. PRBC, FFP, cryoprecipitate and platelets are represented as median requirements per year. IVF represented as mean requirement per year. PRBC – Packed Red Blood Cells / FFP – Fresh Frozen Plasma

Patients presenting with pelvic ring injuries requiring fixation did not typically require blood products, with the overall cohort receiving a median of 0 units of PRBC within the first 24 h (Table 3). Patients within the TS subgroup received a median of 6 (4–9) units PRBC within the first 24 h. The full regression outputs for the overall and TS groups are available in Tables 4 and 5. There was no decrease in 24 h PRBC requirements for the overall cohort (years; IRR = 0.91, 95% CI 0.83–1.01, *p* = 0.066) over the 10-year study period. For the TS subgroup, there was a significant decrease in PRBC requirements over the 10-year period (years; IRR = 0.90, 95% CI 0.85–0.96, *p* = 0.002), with Fig. 2 demonstrating the trends in administration over the study period.

**Table 3:** Crystalloid and blood product requirements by cohort

Outcome	TS (*n* = 47)	Overall (*n* = 144)
24 h PRBC (u)	6 (4–9)	0 (0–4)
24 h FFP (u)	4 (2–7)	0 (0–3.5)
24 h Cryoprecipitate (u)	10 (5–10)	0 (0–5)
24 h Platelets (u)	0 (0–1)	0 (0–0)
24 h IVF (L)	4.5 (3–6)	3.5 (3–5)

Data presented as median with Q1 and Q3 / u – Units / L—Liters

**Table 4:** Multivariate analysis for PRBC transfusion within 24 h of all pelvic ring injured patients

Variable		IRR	CI (95%)	*P*
Year (increasing)		0.91	(0.83–1.01)	0.066
Age (years)		1.00	(0.98–1.01)	0.767
SBP on arrival (mmHg)		0.99	(0.98–0.99)	0.031
Base Excess (mmol/L)		0.90	(0.82–0.98)	0.021
Head Injury (AIS score)				
	Under 3	Ref		
	3 and above	1.84	(1.14–2.98)	0.013
GCS				
	3 to 8	Ref		
	9 to 15	1.60	(1.02–2.49)	0.037
ISS				
	< 15	Ref		
	> 15	0.87	(0.19–4.05)	0.856

*AIS*- abbreviated injury score / *CI*- confidence interval / *GCS*- Glasgow Coma Scale / *IRR*- incidence rate ratio / *ISS* – Injury Severity Score / *PRBC*- packed red bloods cells

**Table 5:** Multivariate analysis for PRBC transfusion within 24 h of pelvic injury for TS subgroup

Variable		IRR	CI (95%)	*P*
Year (increasing)		0.90	(0.84–0.96)	0.002
Time to transfusion (hour)		1.03	(0.89–1.20)	0.690
Age (years)		1.00	(1.00–1.01)	0.377
SBP on arrival (mmHg)		1.00	(0.99–1.01)	0.616
Base Excess (mmol/L)		0.96	(0.92–0.99)	0.013
Head Injury (AIS score)				
	Under 3	Ref		
	3 and above	1.23	(0.86–1.77)	0.259
GCS				
	3 to 8	Ref		
	9 to 15	1.06	(0.73–1.53)	0.774
ISS				
	< 15	Ref		
	> 15	0.61	(0.26–1.46)	0.268

*TS* – Traumatic Shock / *AIS*- abbreviated injury score / *CI*- confidence interval / *GCS*- Glasgow Coma Scale / *IRR*- incidence rate ratio / *ISS* – Injury Severity Score / *PRBC*- packed red bloods cells

Time to first transfusion was not associated with 24 h PRBC requirements for the TS subgroup (hours; IRR = 1.03, 95% CI (0.89–1.20), *p* = 0.690).

#### 24 h Crystalloid, fresh frozen plasma, cryoprecipitate and platelet requirements

A full summary of secondary outcomes is outlined in Tables 3 and 6. Cryoprecipitate administration decreased for the overall cohort (years; IRR = 0.88, 95% CI 0.78–0.99, *p* = 0.035). There was no change in FFP (years; IRR = 0.92, 95% CI 0.82–1.02, *p* = 0.120), platelet (years; IRR = 0.91, 95% CI 0.79–1.05, *p* = 0.216) or IVF (years; IRR = 1.00, 95% CI 0.97–1.02, *p* = 0.719) for the overall cohort.

**Table 6:** Adjusted analysis for intravenous crystalloid and other blood product administration within 24 h of pelvic injury per year of study

Outcome	Overall (*n* = 144)		TS (*n* = 47)	
	IRR (95% CI)	*P* value	IRR (95% CI)	*P* value
24 h FFP (u)	0.92 (0.82–1.02)	0.120	0.92 (0.85–0.99)	0.019
24 h Cryoprecipitate (u)	0.88 (0.78–0.99)	0.035	0.88 (0.81–0.95)	0.001
24 h Platelets (u)	0.91 (0.79–1.05)	0.216	0.89 (0.76–1.04)	0.143
24 h IVF (L)	1.00 (0.97–1.02)	0.719	0.94 (0.90–0.98)	0.004

*TS* – Traumatic Shock / *CI* – Confidence interval / *IRR* – Incidence rate ratio / *FFP* – Fresh frozen Plasma / *IVF* – Intravenous fluids / *u* – units / *L* – liters

For TS patients, there were decreases in cryoprecipitate (years; IRR = 0.88, 95% CI 0.81–0.95, *p* = 0.001), FFP (years; IRR = 0.92, 95% CI 0.85–0.99, *p* = 0.019) and IVF (years; IRR = 0.94, 95%CI 0.90–0.98, *p* = 0.004). There was no change in platelet administration over the study period (years; IRR = 0.89, 95% CI 0.76–1.04, *p* = 0.143). Figure 2 illustrates the trends in FFP, cryoprecipitate, platelet and crystalloid requirements for TS patients over the study period. Full regression outputs for secondary outcomes of overall and TS cohorts are available in supplementary document 2, Tables 2–9.

### Mortality

Throughout the 10-year study period, there were no deaths directly attributed to hemorrhage.

There were five deaths (5/144) during the study period representing an overall mortality rate of 3.5%. Three of the deaths presented in traumatic shock (3/47), resulting in a mortality rate of 6.4% within the TS subgroup.

Causes of death were traumatic brain injury (*n* = 3), Sepsis (*n* = 1) and withdrawal of active care secondary to quality-of-life concerns by family (*n* = 1).

## Discussion

PRBC, cryoprecipitate, FFP and crystalloid administration decreased for the TS subgroup over the 10-year time period, with no acute pelvic hemorrhage-related deaths.

Our institution’s protocol for management of hemodynamically unstable pelvic trauma patients has been in place since 2005 and did not change during the study period. This protocol aims to provide early resuscitation with blood products, minimize crystalloid administration, provide timely hemostatic interventions and early definitive skeletal stabilization.

Large amounts of crystalloid resuscitation have been shown to contribute to dilutional coagulopathy [16]. This ultimately results in worsened hemorrhage, more severe systemic inflammatory response, more frequent organ dysfunctions and worsening mortality [17]. During this 10-year period, intravenous crystalloid administration within the first 24 h decreased among TS patients at a rate of approximately 6% per year. Clinically, this equates to a 48% decrease in crystalloid administration at the end of the study period compared to the beginning. This decrease in crystalloid administration highlights the increased adherence to the resuscitation protocol, contributing to decreases in packed cell and clotting factor requirements.

**Fig. 3:**
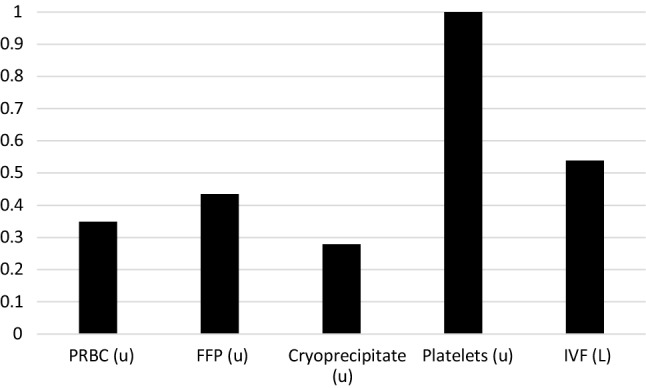
Likelihood of receiving 1 product at the end of the 10-year study period compared to the beginning for TS subgroup. TS – Traumatic Shock / PRBC – Packed Red Blood Cells / FFP – Fresh Frozen Plasma / IVF – Intravenous Fluid /*U* = Units / *L* = Liters

Administration of blood products over crystalloid reduces the risk of dilutional coagulopathy thereby improving hemostasis and contributing to decreased overall blood product requirements [16]. As Table 3 highlights, TS patients in this study received large transfusion volumes with a transfusion ratio of approximately 1:1:2. Over the 10-year study period, as adherence improved, PRBC requirements decreased by approximately 10% per year, equating to a 65% decrease in PRBC administration at the end of the 10-year period compared to the beginning. Figure 3 demonstrates the likelihood of blood and factor administration at the end of the study period compared to the beginning with Fig. 2 demonstrating the trends in administration over the study period.

There is little high-quality evidence assessing the impact of early transfusion on overall transfusion requirements. A recent systematic review by Turenhout (2019) did not identify consistent benefits of early transfusion among civilian trauma patients, noting the absence of high-quality research [18]. Our analysis did not identify an association between earlier initial transfusion and decreased 24 h PRBC requirements among TS patients (hours; IRR = 1.03, 95% CI 0.89–1.20, *p* = 0.690), consistent with Turenhout findings. Interestingly, delayed time to transfusion was associated with increased IVF administration (IRR = 1.08, 95% CI 1.01–1.18, *p* = 0.048) further highlighting the importance of early hemostatic resuscitation, as the risk of IVF-induced dilutional coagulopathy is reduced with decreased crystalloid administration [16].

The need for urgent hemostatic procedures (Angiography and Peritoneal packing) has been reported to decrease since the adoption of improved resuscitation strategies [8]. Gaski (2016) reviewed outcomes over the first five years of a protocols implementation, identifying that modern protocols were associated with lower rates of angioembolization and pre-peritoneal pelvic packing, however, were also associated with increased platelet and plasma requirements [8]. The decreases in transfusion requirements among unstable pelvic trauma patients observed in this study highlight the improvements in outcomes that occurs as a protocol matures, following on from the benefits of the implementation of such protocols.

Pelvic fracture mortality ranges from 2.8% in all-comers to 32% for patients presenting in shock, with hemorrhage the most common cause [19–21]. To identify all instances of pelvic fracture-related mortality, our institutions mortality review panel data were analyzed. During this time period, 5 patients with confirmed or suspected pelvic injury died within the emergency department, with all patients having pre-hospital cardiac arrest and receiving CPR on arrival. For patients with pelvic injuries presenting alive to our institution, 5 patients died with no deaths directly attributed to hemorrhage over the 10-year period. We attribute this lack of hemorrhage-related mortality to our mature resuscitation protocol, immediate availability of blood products and access to trauma and interventional specialists for acute definitive management. Despite the severely injured nature of TS patients (median ISS of 38), the overall mortality rate was 6.4%, with the majority of deaths secondary to traumatic brain injuries and no deaths directly attributed to hemorrhage.

This study is a retrospective review of a prospectively collected institutional pelvic trauma-specific database. As most information was retrieved from this prospective database, the authors believe this provides better quality data than typical retrospective studies; however, this study is still limited by its retrospective nature and small sample size. Transfusion information and initial hemodynamics were unable to be accurately recorded for patients retrieved from other institutions and they were therefore unable to be included in this study. Administration of platelets and clotting factors occurs as part of the MTP in the emergency department; while time to commencement of MTP was typically documented, the exact time of commencement of individual products (FFP, Cryoprecipitate, Platelets) was inconsistently documented and therefore was unable to be included. Despite the limitations, the findings of our study hold external validity among trauma centers that regularly manage pelvic trauma as our pelvic fracture protocol is based on modern resuscitation principles and our demographics are similar to previously reported populations [15, 22].

## Conclusion

Over this 10-year time period, there were no hemorrhage-related deaths among patients presenting with unstable pelvic fractures. Crystalloid and transfusion requirements decreased among patients presenting with traumatic shock. Future studies on improvements in massive transfusion protocols should be reported in the context of crystalloid administration.

## Supplementary Information

Below is the link to the electronic supplementary material.

Supplementary file1 (DOCX 34 KB)

Supplementary file2 (DOCX 33 KB)
