# Supplementary material for: Major Pelvic Ring Injuries: Fewer Transfusions Without Deaths from Bleeding During the Last Decade
Source: World J Surg. 2023 Jan 17;47(5):1136–43. doi: 10.1007/s00268-023-06897-7 (PMC10070203; doi:10.1007/s00268-023-06897-7)
Supplement: Supplementary file 1 — Supplementary file1 (DOCX 34 KB) [file 268_2023_6897_MOESM1_ESM.docx]

**Supplementary Table 1:** Number of missing data-points per variable

| Variable | Overall Cohort (n=144) | TS (n=47) |
| --- | --- | --- |
| Age | 0 (0%) | 0 (0%) |
| Gender | 0 (0%) | 0 (0%) |
| ISS | 0 (0%) | 0 (0%) |
| Hemodynamically Unstable | 0 (0%) | 0 (0%) |
| Head AIS | 0 (0%) | 0 (0%) |
| GCS | 0 (0%) | 0 (0%) |
| Base Excess | 7 (5%) | 0 (0%) |
| Lactate | 8 (6%) | 0 (0%) |
| pH | 6 (4%) | 0 (0%) |
| SBP (arrival) | 0 (0%) | 0 (0%) |
| 24hr PRBC (u) | 9 (6%) | 1 (2%) |
| 24hr FFP (u) | 9 (6%) | 1 (2%) |
| 24hr Cryoprecipitate (u) | 9 (6%) | 1 (2%) |
| 24hr Platelets (u) | 9 (6%) | 1 (2%) |
| 24hr IVF (L) | 0 (0%) | 0 (0%) |
| Mortality | 0 (0%) | 0 (0%) |

| **Supplementary Table 2.** Multivariate analysis for FFP transfusion within 24 hours of pelvic injury for the overall cohort | | | |
| --- | --- | --- | --- |
| Variable | IRR | CI (95%) | P |
| Year (increasing) | 0.92 | (0.82-1.02) | 0.120 |
| Age (years) | 1.00 | (0.98-1.02) | 0.656 |
| SBP on arrival (mmHg) | 0.99 | (0.97-1.00) | 0.086 |
| Base Excess (mmol/L) | 0.89 | (0.79-0.99) | 0.037 |
| Head Injury (AIS score) |  |  |  |
| Under 3 | Ref. |  |  |
| 3 and above | 1.19 | (0.64-2.20) | 0.585 |
| GCS |  |  |  |
| 3 to 8 | Ref. |  |  |
| 9 to 15 | 2.06 | (1.16-3.65) | 0.013 |
| ISS |  |  |  |
| <15 | Ref. |  |  |
| >15 | 0.49 | (0.08-3.17) | 0.116 |
| AIS- abbreviated injury score / CI- confidence interval / GCS- Glasgow Coma Scale / IRR- incidence rate ratio / FFP – Fresh Frozen Plasma / ISS – Injury Severity Score | | | |

| **Supplementary Table 3.** Multivariate analysis for Cryoprecipitate transfusion within 24 hours of pelvic injury for the overall cohort | | | |
| --- | --- | --- | --- |
| Variable | IRR | CI (95%) | P |
| Year (increasing) | 0.88 | (0.78-0.99) | 0.035 |
| Age (years) | 1.00 | (0.98-1.01) | 0.591 |
| SBP on arrival (mmHg) | 0.97 | (0.96-0.99) | <0.001 |
| Base Excess (mmol/L) | 0.88 | (0.78-0.98) | 0.022 |
| Head Injury (AIS score) |  |  |  |
| Under 3 | Ref. |  |  |
| 3 and above | 1.23 | (0.61-2.46) | 0.562 |
| GCS |  |  |  |
| 3 to 8 | Ref. |  |  |
| 9 to 15 | 2.32 | (1.22-4.41) | 0.010 |
| ISS |  |  |  |
| <15 | Ref. |  |  |
| >15 | 0.48 | (0.08-2.81) | 0.414 |
| AIS- abbreviated injury score / CI- confidence interval / GCS- Glasgow Coma Scale / IRR- incidence rate ratio / ISS – Injury Severity Score | | | |

| **Supplementary Table 4.** Multivariate analysis for Platelet transfusion within 24 hours of pelvic injury for the overall cohort | | | |
| --- | --- | --- | --- |
| Variable | IRR | CI (95%) | P |
| Year (increasing) | 0.91 | (0.79-1.05) | 0.216 |
| Age (years) | 1.01 | (0.99-1.03) | 0.405 |
| SBP on arrival (mmHg) | 0.99 | (0.97-1.01) | 0.325 |
| Base Excess (mmol/L) | 0.89 | (0.83-0.96) | 0.002 |
| Head Injury (AIS score) |  |  |  |
| Under 3 | Ref. |  |  |
| 3 and above | 2.54 | (0.86-7.47) | 0.090 |
| GCS |  |  |  |
| 3 to 8 | Ref. |  |  |
| 9 to 15 | 1.51 | (0.61-3.80) | 0.374 |
| ISS |  |  |  |
| <15 | Ref. |  |  |
| >15 | 0.90 | (0.13-6.42) | 0.918 |
| AIS- abbreviated injury score / CI- confidence interval / GCS- Glasgow Coma Scale / IRR- incidence rate ratio / ISS – Injury Severity Score | | | |

| **Supplementary Table 5.** Multivariate analysis for IVF transfusion within 24 hours of pelvic injury for the overall cohort | | | |
| --- | --- | --- | --- |
| Variable | IRR | CI (95%) | P |
| Year (increasing) | 1.00 | (0.97-1.02) | 0.719 |
| Age (years) | 1.00 | (0.99-1.00) | 0.183 |
| SBP on arrival (mmHg) | 1.00 | (1.00-1.00) | 0.734 |
| Base Excess (mmol/L) | 0.98 | (0.96-1.00) | 0.049 |
| Head Injury (AIS score) |  |  |  |
| Under 3 | Ref. |  |  |
| 3 and above | 1.04 | (0.89-1.23) | 0.610 |
| GCS |  |  |  |
| 3 to 8 | Ref. |  |  |
| 9 to 15 | 1.05 | (0.83-1.34) | 0.674 |
| ISS |  |  |  |
| <15 | Ref. |  |  |
| >15 | 1.45 | (1.12-1.87) | 0.005 |
| AIS- abbreviated injury score / CI- confidence interval / GCS- Glasgow Coma Scale / IRR- incidence rate ratio / IVF – Intravenous Fluid (crystalloid) / ISS – Injury Severity Score | | | |

| **Supplementary Table 6.** Multivariate analysis for FFP transfusion within 24 hours of pelvic injury for TS subgroup | | | |
| --- | --- | --- | --- |
| Variable | IRR | CI (95%) | P |
| Year (increasing) | 0.92 | (0.85-0.99) | 0.019 |
| Time to transfusion (hour) | 1.00 | (0.77-1.29) | 0.997 |
| Age (years) | 1.00 | (0.99-1.01) | 0.657 |
| SBP on arrival (mmHg) | 1.00 | (0.99-1.01) | 0.381 |
| Base Excess (mmol/L) | 0.95 | (0.89-1.01) | 0.114 |
| Head Injury (AIS score) |  |  |  |
| Under 3 | Ref. |  |  |
| 3 and above | 0.90 | (0.52-1.56) | 0.708 |
| GCS |  |  |  |
| 3 to 8 | Ref. |  |  |
| 9 to 15 | 1.09 | (0.63-1.86) | 0.760 |
| ISS |  |  |  |
| <15 | Ref. |  |  |
| >15 | 0.34 | (0.06-1.81) | 0.205 |
| TS – Traumatic Shock / AIS- abbreviated injury score / CI- confidence interval / GCS- Glasgow Coma Scale / IRR- incidence rate ratio / FFP- Fresh Frozen Plasma / ISS – Injury Severity Score | | | |

| **Supplementary Table 7.** Multivariate analysis for Cryoprecipitate transfusion within 24 hours of pelvic injury for TS subgroup | | | |
| --- | --- | --- | --- |
| Variable | IRR | CI (95%) | P |
| Year (increasing) | 0.88 | (0.81-0.95) | 0.001 |
| Time to transfusion (hour) | 0.94 | (0.76-1.15) | 0.518 |
| Age (years) | 1.00 | (0.99-1.01) | 0.864 |
| SBP on arrival (mmHg) | 0.99 | (0.98-1.00) | 0.039 |
| Base Excess (mmol/L) | 0.96 | (0.92-1.00) | 0.060 |
| Head Injury (AIS score) |  |  |  |
| Under 3 | Ref. |  |  |
| 3 and above | 0.81 | (0.52-1.25) | 0.335 |
| GCS |  |  |  |
| 3 to 8 | Ref. |  |  |
| 9 to 15 | 1.06 | (0.71-1.57) | 0.786 |
| ISS |  |  |  |
| <15 | Ref. |  |  |
| >15 | 0.44 | (0.17-1.17) | 0.100 |
| TS – Traumatic Shock / AIS- abbreviated injury score / CI- confidence interval / GCS- Glasgow Coma Scale / IRR- incidence rate ratio / ISS – Injury Severity Score | | | |

| **Supplementary Table 8.** Multivariate analysis for Platelet transfusion within 24 hours of pelvic injury for TS subgroup | | | |
| --- | --- | --- | --- |
| Variable | IRR | CI (95%) | P |
| Year (increasing) | 0.89 | (0.76-1.04) | 0.143 |
| Time to transfusion (hour) | 1.01 | (0.64-1.61) | 0.954 |
| Age (years) | 1.01 | (0.99-1.03) | 0.280 |
| SBP on arrival (mmHg) | 1.00 | (0.98-1.02) | 0.910 |
| Base Excess (mmol/L) | 0.93 | (0.86-1.00) | 0.054 |
| Head Injury (AIS score) |  |  |  |
| Under 3 | Ref. |  |  |
| 3 and above | 1.98 | (0.62-6.32) | 0.250 |
| GCS |  |  |  |
| 3 to 8 | Ref. |  |  |
| 9 to 15 | 1.02 | (0.38-2.74) | 0.965 |
| ISS |  |  |  |
| <15 | Ref. |  |  |
| >15 | 0.73 | (0.90-5.91) | 0.768 |
| TS – Traumatic Shock / AIS- abbreviated injury score / CI- confidence interval / GCS- Glasgow Coma Scale / IRR- incidence rate ratio / ISS – Injury Severity Score | | | |

| **Supplementary Table 9.** Multivariate analysis for IVF transfusion within 24 hours of pelvic injury for TS subgroup | | | |
| --- | --- | --- | --- |
| Variable | IRR | CI (95%) | P |
| Year (increasing) | 0.94 | (0.90-0.98) | 0.004 |
| Time to transfusion (hour) | 1.08 | (1.00-1.18) | 0.048 |
| Age (years) | 1.00 | (0.99-1.00) | 0.246 |
| SBP on arrival (mmHg) | 1.00 | (1.00-1.01) | 0.393 |
| Base Excess (mmol/L) | 0.99 | (0.97-1.02) | 0.393 |
| Head Injury (AIS score) |  |  |  |
| Under 3 | Ref. |  |  |
| 3 and above | 1.03 | (0.78-1.35) | 0.856 |
| GCS |  |  |  |
| 3 to 8 | Ref. |  |  |
| 9 to 15 | 1.05 | (0.80-1.39) | 0.719 |
| ISS |  |  |  |
| <15 | Ref. |  |  |
| >15 | 1.70 | (1.00-2.87) | 0.048 |
| TS – Traumatic Shock / AIS- abbreviated injury score / CI- confidence interval / GCS- Glasgow Coma Scale / IRR- incidence rate ratio / IVF- intravenous fluid (crystalloid) / ISS – Injury Severity Score | | | |
